# Applications of Prolamin-Based Edible Coatings in Food Preservation: A Review

**DOI:** 10.3390/molecules28237800

**Published:** 2023-11-27

**Authors:** Shuning Zhang, Yongyan Kuang, Panpan Xu, Xiaowei Chen, Yanlan Bi, Dan Peng, Jun Li

**Affiliations:** College of Food Science and Engineering, Henan University of Technology, Zhengzhou 450001, China; 2021930541@stu.haut.edu.cn (S.Z.); k2020920035@stu.haut.edu.cn (Y.K.); xupanpan@stu.haut.edu.cn (P.X.); fexwchen@haut.edu.cn (X.C.); byl@haut.edu.cn (Y.B.); pengdan@haut.edu.cn (D.P.)

**Keywords:** prolamins-based edible coating, coating preservation techniques, modifications

## Abstract

Foods are susceptible to deterioration and sour due to external environmental influences during production and storage. Coating can form a layer of physical barrier on the surface of foods to achieve the purpose of food preservation. Because of its good barrier properties and biocompatibility, prolamin-based film has been valued as a new green and environment-friendly material in the application of food preservation. Single prolamin-based film has weaknesses of poor toughness and stability, and it is necessary to select appropriate modification methods to improve the performance of film according to the application requirements. The practical application effect of film is not only affected by the raw materials and the properties of the film itself, but also affected by the selection of preparation methods and processing techniques of film-forming liquid. In this review, the properties and selection of prolamins, the forming mechanisms and processes of prolamin-based coatings, the coating techniques, and the modifications of prolamin-based coatings were systematically introduced from the perspective of food coating applications. Moreover, the defects and deficiencies in the research and development of prolamin-based coatings were also reviewed in order to provide a reference for the follow-up research on the application of prolamin-based coatings in food preservation.

## 1. Introduction

Food products may deteriorate during production, processing, distribution, transportation, and storage due to physical actions, chemical reactions, and microbial contamination. Fruits and vegetables will cause abrasions or tissue damage due to physical shock and vibration, and are very sensitive to the storage environment (e.g., ethylene, oxygen, water vapor are the main causes of post-harvest quality deterioration in fruits and vegetables [1]). High-fat products (such as fish, meat, sausages, nuts, etc.) are prone to be oxidized due to the contact of oils and fats with external oxygen and moisture, as well as microbial contamination and the rapid multiplication of microbes (e.g., Pseudomonas, Acinetobacter, Brochothrix, Shewanella, Aeromonas, and others are usually considered the dominant communities in chilled meat and poultry storage under aerobic conditions [2]), forming the rancid odor and even substances that are harmful to the human body [3]. Foods (such as sultanas, breakfast cereals, biscuits, fruit slices, etc.) that need to maintain low moisture activity would lose their original crispness or dryness due to water migration, resulting in poor organoleptic properties. At present, food packaging is considered to be one of the effective means to address those issues [4], and thus plays an important role in the preservation, circulation, and sales of foods. Traditional food packaging materials are mostly derived from petroleum-based chemical intermediates. However, the extensive application of petroleum-based polymeric materials is gradually deteriorating the ecosystem and living environment. Therefore, topics are being shifted to the research, development, and application of environment-friendly and naturally polymeric materials.

Coating is a novel packaging technique, in which natural edible macromolecules (e.g., polysaccharides, proteins, etc.) are used as the raw materials and cured on the food surface by impregnation, spraying, or coating to form a tightly structured, protective, and semi-permeable coated film (in this review, the term coating only referred to coated film, while the term film referred to both individual film and coated film) [5]. Coating can give continuous protection to foods when there is no outer packaging or the outer packaging is broken. As an effective, safe, environmentally-friendly, low-cost, and easy-to-use technique, coating has been widely used in fruit and vegetable preservation, aquatic product preservation, nut preservation, drug coating, and many other fields [6,7].

There are various types of edible films, which can be broadly classified into polysaccharide-based, protein-based, lipid-based, and composite edible films according to their raw materials. Edible films made from different raw materials have different properties and characteristics in terms of mechanical strengths, water and gas barrier capabilities, storage stability, etc. Thus, comprehensive consideration should be taken when selecting edible films and corresponding raw materials such as properties of both edible films and food products to be filmed/coated as well as application environments. Polysaccharide-based films (e.g., chitosan, starch and its derivatives, cellulose and its derivatives, sodium alginate, pullulan polysaccharide, pectin, etc.) are generally homogeneous and transparent and have good mechanical properties and antibacterial properties as well as high carbon dioxide and oxygen permeability. Therefore, polysaccharide-based films are commonly used for protecting meat from microbial growth [8], and fruit and vegetable preservation [9]. Some polysaccharide-based films are highly hydrophilic to the extent that the films are weak in water vapor transmission rate and water resistance [10]. These films are commonly used for manufacturing water-soluble packaging bags (such as seasoning powder, coffee powder packaging, etc.) [11]. Lipid-based edible films are mainly based on waxes, fatty acids, resins, etc., and usually have high water resistance because of the strong hydrophobicity of aliphatic carbon chains. However, lipid-based films formed by oils and fats alone have only few applications due to their poor mechanical properties. Typically, oils and fats can be used as additives to laminate or form emulsions with other materials in order to improve the water resistance, water vapor barrier, transparency, elongation, etc., of composite films [12]. In terms of coating preservation of intact fruits, lipid-based materials are used widely, which can not only block water evaporation and keep fruits fresh, but also make the surface of fruits glossy and bring better sensory characteristics to consumers. However, lipid-based coatings tend to have a waxy taste when consumed directly. Compared with polysaccharide-based and lipid-based edible films, protein-based edible films have good gas barrier properties, mechanical properties, and palatability, and thus are often used for foods that are prone to be oxidized during storage. Wang et al. [13] used gluten powder (WG) and soybean protein isolate (SPI) as the main raw materials to prepare the coating solution and coated it on the surface of peanut and walnut kernels by spraying. Peanuts wrapped with WG coating, SPI coating, and mixed WG/SPI coating had 1, 2.5, and 1.8 (mg KOH/g) lower acid values and 20, 60, and 50 (meq/kg) lower peroxide values than the non-wrapped control group after 100 days of storage, which well controlled the oil deterioration of the kernels during storage. Proteins are large molecules with a specific amino acid (AA) sequence and molecular structure, and their physicochemical properties largely depend on the AA composition, the arrangement of AA substituents, and their relative number in the polymer chain [14]. The high content of hydrophilic AAs in proteins results in poor moisture barrier properties in most protein-based films.

Prolamins are a large category of grain seed storage protein (most storage proteins can be divided into four categories: 11S globulins, 7S globulins, 3S albumins, and prolamins), which are deposited in discrete protein bodies within seed cells prior to germination and provide elements (such as nitrogen, carbon, and sulfur) required for growth and development during germination [15]. Prolamins belong to a group of proteins and prolamin-based films are thus similar to protein-based films. Compared to polysaccharide- and lipid-based films, prolamin-based films have an outstanding gas barrier and good palatability. Compared to other proteins, most prolamins contain a relatively high ratio of hydrophobic AA. Therefore, prolamin-based films are more hydrophobic and less soluble in water. Due to the good water vapor barrier and gas barrier properties as well as green safety and biocompatibility, prolamin-based films are widely used in food and drug packaging, such as preservation of fruits and vegetables [16], meats [17], nuts [18], and the slow release of drugs [19]. However, single-prolamin-based films usually have hard and brittle texture, poor plasticity, high sensitivity to ambient temperature and humidity (swelling easily occurs in high humidity environments), and poor stability. To meet the application needs, researchers improve the film properties by physical and chemical means or by compounding other materials. At present, the research on prolamin-based films mainly focuses on the influences of film-forming conditions and modification methods on the properties of the resulting films, which can have a straightforward effect on the improvement of films applied for outside packaging. When prolamin-based coatings are directly coated on the surface of foods for fresh-keeping preservation, the final application effects will be largely affected by the selection of prolamins as the raw materials, the preparation conditions of coating solution, the pattern of coating processes, the surface properties of foods, and the interaction between coating solution and foods. Since prolamin-based coating has direct contact with the coated foods and undergoes simultaneous storage with the coated foods, and it needs to be edible, there are more restrictive requirements for coating development compared to film development. However, most of the current reports on prolamin-based films mainly focus on how to prepare films with better properties by modification of films, which may be applied to food overwrap or industrial applications. Reports on edible coatings mainly focus on the effects of coating preservation on food quality enhancement. A review has been presented on the preparation and application of starch-based edible coatings [20], but the review on prolamin-based coatings has not been found. The objectives of the work were to review the raw material analysis, coating-forming mechanisms, processing processes, and modification methods of prolamin coatings from the perspective of food coating preservation, and summarize the possible influencing factors and the existing shortcomings, in order to provide scientific references for subsequent studies on the application of prolamin-based coatings in food preservation.

## 2. Preparation of Prolamin-Based Coatings

### 2.1. Selection of Prolamins

The structure and properties of prolamins vary largely depending on crop variety, as reflected by their percentages in total proteins, AA composition, disulfide bond content, protein structure, etc. The differences in physical and chemical properties make different types of prolamins have different functional properties. For example, the surface properties of prolamins such as hygroscopicity, solubility, dispersibility, foaming, emulsification, and water and oil retention are mainly influenced by AA composition, distribution, folding pattern, and the hydrophilic/hydrophobic steric characteristics; the hydrodynamic properties of prolamins such as viscosity, gelation, and organization are mainly influenced by the molecular weight, shape, and molecular flexibility of prolamins [21]. In practical applications, appropriate materials should be selected according to changes in applied environments and demands. At present, the prolamins that are widely used in the field of food coating and film preservation mainly include hordein, gliadin, zein, and kafirin, among which zein is the most extensively studied. This is because (1) maize has a large advantage in production over other cereals (maize 40%, wheat 28%, rice 29%, sorghum 4%); (2) the production technique of zein is relatively mature, and its production has been commercialized. Most prolamins contain a relatively high ratio of glutamine and proline. The four prolamins have significant homology and similarity based on the sequence of AAs [22]. A detailed study on the different species of proteins will benefit better understanding the differences among them and thus identifying their strengths and weaknesses and scopes in applications.

The formation of prolamin-based films mainly depends on non-covalent forces, including hydrogen bonds, hydrophobic interactions, van der Waals forces, and other forces, to link the polypeptide chains together. Therefore, the AA composition of a protein largely determines the basic properties of protein-based films. Cao [23] determined the AA composition of lab-extracted hordein and compared it with that of gliadin, zein, and kafirin. The results showed that compared to zein, hordein contains more glutamic acid and proline, but less alanine and leucine. It is observed that hordein is less hydrophobic and contains more intermolecular hydrogen bonds than zein since glutamic acid is usually involved in hydrogen bond formation and alanine and leucine are non-polar AAs with aliphatic side chains. This was confirmed by subsequent experiments where hordein had better oil holding capacity, emulsification, and emulsification stability. The gliadin-based film is sensitive to moisture in the environment and has poor water barrier performance, but has good oxygen barrier ability in the case of low-relative humidity. The AA compositions of the four prolamins are shown in Table 1. The AA compositions of hordein and gliadin are similar, while the AA compositions of zein and kafirin are similar. Shewry [15] compared zein with kafirin from the perspectives of biochemistry, molecular biology, and biophysics, and found that the molecular weight and structure of the two prolamins are similar. However, kafirin is more hydrophobic and less digestible than zein [24], which makes kafirin-based film more stable with superior gas and water vapor barrier properties and stronger protective functions in packaging applications [25]. Thus, kafirin is being developed as a potential alternative to zein in film-forming applications. Qazanfarzadeh et al. [26] prepared secalin film with zein film as comparison, and found that secalin film showed lower contact angle values and higher moisture content, solubility, and swelling index than zein film due to the fact that zein has a higher content of hydrophobic AAs than secalin.

In some cysteine-rich prolamins, covalent and disulfide bonds are formed between or within the peptide chains, thus affecting the film properties. The part of wheat gluten that can be extracted by aqueous ethanol is called gliadin, and the other part that cannot be dissolved in aqueous ethanol is called glutenin. The AA compositions of them are basically similar, while disulfide bonds in wheat gliadin are intramolecular and in glutenin are intermolecular [27]. Hernández-Muñoz et al. [28] found that gliadin-based films rupture once exposed to water, while glutenin-based films still remain intact after soaking in water for 24 h. The water stability of gliadin-based films is significantly improved by adding cysteine as a cross-linking agent, which increases the intermolecular force and disulfide bond content of the resulting films. This indicates that the strengths of intermolecular forces in prolamin-based films have a significant effect on the properties of the resulting films. In the modifications of edible films, increasing the intermolecularly cross-linking degree of disulfide bonds can be used to improve film stability [29].

Different secondary structures of prolamins also affect the properties of the resulting films. Oliviero et al. [30] found that zein with high contents of α-helical structure makes zein-based films have stronger mechanical strength than those with high contents of β-sheet. Belton et al. [31] suggested that high contents of β-sheet lead kafirin to clump in the film-forming solution, which affects the film-forming effect and makes the resulting films uneven. Therefore, prolamin aggregation should be minimized and native α-helical structure maximized during extraction in order to obtain a material suitable to provide the best film quality.

### 2.2. Extraction of Prolamins

Prolamins can be further divided into four parts according to the molecular weight, which are usually represented by α-, β-, γ-, and δ-components. The four components have differences in AA composition, structure, sequence, and disulfide bond content. At present, a large number of detailed studies and reviews [31,32,33] have proved that the ratio of the four components affects the functional properties of the whole prolamins. Due to polar differences, the four components have varying degrees of sensitivity to the extracting solvents. The selection of extracting solvents and methods largely affects the content of each component in the finished products, which in turn affects the final application effects. The main processes of prolamin extraction can be roughly divided into two steps.

#### 2.2.1. Extraction

Grains as the raw materials are extracted based on the feature that prolamin can dissolve in organic solvents. Moreover, to save the production cost, industrial by-products are usually used as the raw materials. For example, corn gluten meal, a by-product from the wet milling process of corn starch production, contains a large amount of zein and is inexpensive, making it an ideal raw material for commercial zein production [34]; kafirin can be produced from some by-products of milling or brewing industries [35,36]. Due to the particularity of the application of food coatings, it is necessary to consider both the extracting ability and the food compatibility of extracting solvents. For example, aqueous ethanol, acetone, and isopropanol all can be used to extract zein from corn gluten meal. Currently, the most common commercial extracting processes for zein are: corn gluten meal is extracted once with 88% (*w*/*w*) aqueous isopropanol containing 0.25% NaOH at 55~65 °C; the extracting solution is cooled down to 10~−20 °C to precipitate zein; and the extracting and cooling processes are repeated alternately to improve the purity of zein. In addition to aqueous ethanol, Selling and Woods [37] used acetic acid as the extracting solvent to extract a large amount of alcohol solubles from corn gluten meal. Sodium dodecyl sulfate polyacrylamide gel electrophoresis showed that the protein bands from acetic acid-extracted alcohol solubles are similar to those extracted from commercial zein and 80% ethanol-extracted zein. The mechanical properties of films prepared from acetic acid-extracted zein extractives are superior to those prepared from commercial zein. However, this extracting method needs to use expensive solvents and the resulting zein has a pungent and sour smell, which limits its application. Reducing agents are reported to have the ability of destroying the disulfide bonds in proteins during extraction, thereby improving the solubility of proteins in the extracting solvents and the extracting yield [38]. However, Schober et al. [39] found that the addition of reducing agents during extraction reduces the proportion of α-zein in the finished product; β- and γ-zeins form insoluble matters in the film-forming solution when casting films, which affects the flatness of the resulting films. The hydrophobicity of β- and γ-zein is slightly lower than that of α-zein. The high proportion of α-zein benefits from producing more films. Thus, the addition of reducing agents may affect the application performance of coating.

#### 2.2.2. Separation and Purification

The filtrate is collected by filtering and/or centrifuging the extracting solution, followed by adding excessive cold water to dilute the concentration of organic solvent in the extracting solution or cooling it down to make prolamins precipitated out. After filtration and/or centrifugation, collected prolamins are vacuum-dried and ground to achieve the final product. The purity of prolamins can be improved by repeating the extraction and separation operations or adjusting the solvent concentration. Anderson and Lamsal [40] improved the commercial extraction processes of zein and found that adding acetone or anhydrous ethanol into the extracting solution system before cooling down for zein precipitation benefits from removing partial β- and γ-zein and increasing the proportion of α-zein in the final product. At present, the main blocks faced by the large-scale production of prolamin are still the high extracting cost, low extracting yield, and unstable quality (such as protein content, color intensity, and gel properties in ethanol solution).

### 2.3. Preparation of Coating Solutions

Currently, the common methods of casting membranes for prolamin-based films include solvent casting (bench- and continuous casting), extrusion (slit-die- and blown-film extrusion), electrospinning, and calendaring. Amongst them, solvent casting, especially continuous casting, is considered easy and the most suitable method for manufacturing prolamin-based coating. Solvent casting needs to dissolve or disperse prolamins in a suitable solvent (heating if necessary) and apply the solution onto the surface of the object to be coated, then solvents are evaporated at high or ambient temperatures. During solvent evaporation, the concentration of the polymer in the medium increases, and the polymer is induced to bond and form a three-dimensional network structure, namely film.

In the early 1940s, researchers had conducted comprehensive and extensive studies on the solubility of zein in various solvents (including primary solvents, binary solvents, and ternary solvents). In addition to the commonly used aqueous ethanol solution, aqueous isopropanol solution, aqueous acetone solution, lactic acid, and glacial acetic acid all can be used as solvents for prolamin film-forming solutions [39]. Different organic solvents have different polarities, and their main functional groups are hydroxyl, carboxyl, and carbonyl groups, respectively, which carry different electronegativities, thus affecting the folding and unfolding of hydrophobic long chains of prolamins as well as their state of dissolution and dispersion in solution [41]. Nathania et al. [42] investigated the effect of solvent polarity on the film properties of kafirin and showed that with reduced solvent polarity, the α-helical of kafirin in the solvent tends to increase but the water contact angle increases. The tensile strength and Young’s modulus do not show any specific trend with varying solvent polarity within the same type of solvent, but kafirin-*t*-butanol films give higher tensile strength and elongation at break compared to kafirin-ethanol films.

Good solubility and volatility are essential for the selection of solvents used for film-forming solutions. When preparing the film-forming solution, prolamin should be uniformly dispersed in the solution to eliminate the inhomogeneity and even phase separation in the resulting films. The production of a continuous and intact film requires high binding strengths among protein molecules to ensure the rapid binding between adjacent polymer molecules and the disappearance of the boundary layer of films. In the case of slower solvent evaporation, protein molecules clump and precipitate out before forming a continuous film matrix due to low cohesive strengths. For example, Taylor et al. [25] reported that when using 55% (*w*/*w*) aqueous isopropanol to cast kafirin films at 40 °C, the resulting films are opaque and split into many pieces; when using 70% (*w*/*w*) aqueous ethanol at 40 °C or glacial acetic acid at 25 °C, intact films are achieved.

**Figure 1:**
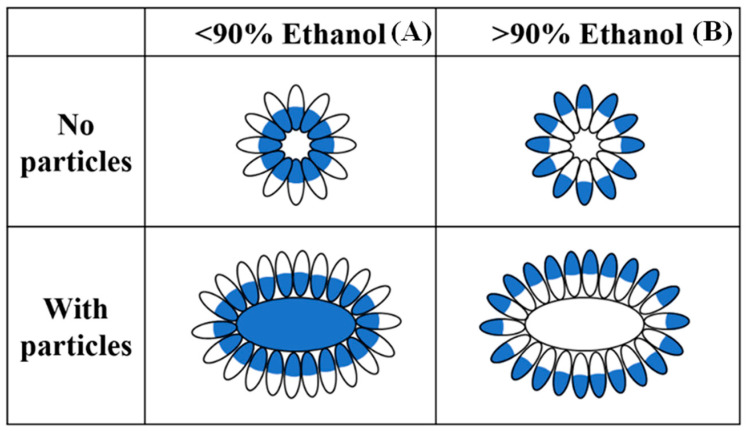
Schematic diagram of micellar structure changes of zein in ethanol solution ((**A**): micellar structure of zein in <90% ethanol, (**B**): micellar structure of zein in >90% ethanol).

Aqueous ethanol is the most commonly used solvent to cast prolamin-based films, since most prolamins can dissolve in 40~90% aqueous ethanol. An appropriate concentration of ethanol can not only enhance the solubilization of prolamins, but also the intermolecular forces between protein molecular chains as well as proteins and other components, easily forming stronger and more dense films [43]. A previous study reported by Kim and Xu [44] found that the micellar structure of zein aggregates is inverted in about 90% aqueous ethanol (Figure 1). In other words, the hydrophilic part of the micelle-like structure exposes to the solvent medium when the ethanol concentration is <90% (Figure 1A), and the surface charges of the particles repel each other at this time, thereby forming a cloudy solution; while the hydrophobic part exposes to the solvent medium when the ethanol concentration is >90%, the surface charges of the particles attract each other at this time, thereby aggregating and precipitating (Figure 1B). Yin et al. [45] superimposed the zein coating on the sodium caseinate-based film to modify the hydrophobicity of the resulting film, and found that the water vapor transmission rate of the modified films after micelle inversion decreased from 4.75 to 3.95 compared to the films without micelle inversion, and the modified film after micelle inversion significantly improved hydrophobicity but had reduced surface smoothness in comparison with the unmodified film. Although no literature has been found to prove whether or not other prolamins have similar properties, the micelle inversion method provides research ideas for the hydrophobic modification of prolamin-based films and prolamin particles.

**Figure 2:**
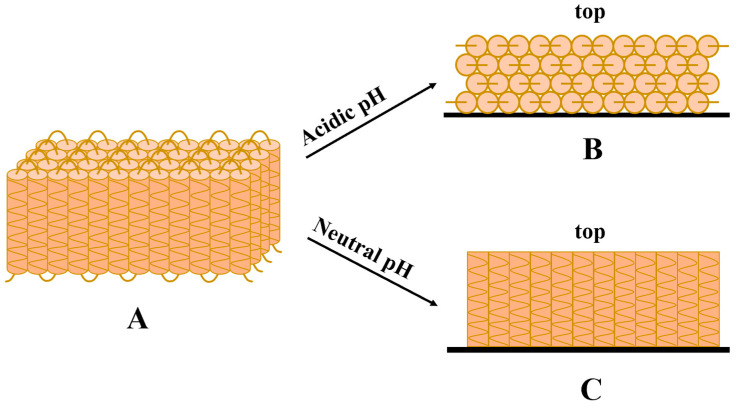
Schematic diagram of molecular arrangement of zein in acidic and neutral environment ((**A**): structural model of α-zein; (**B**): structural model of α-zein under acidic conditions; (**C**): structural model of α-zein under neutral conditions).

Organic acids can donate and accept electrons, form hydrogen bonds between them and prolamins, and dissolve prolamins at lower heating temperatures (or normal temperatures), and form thinner and more uniform films [25]. Films made with alkaline solutions may have better mechanical strengths than those made with acidic solutions [46]. Matsushima et al. [47] proposed a hypothesis for the structural model of α-zein: it is composed of multiple α-helical repeating sequence units arranged side by side and connected in series (Figure 2A). Based on this model, Shi et al. [48] analyzed the structures of zein-based films formed with different solvents and concluded that the conformational reorganization of zein molecules varies during solvent evaporation, largely depending on the pH value of the system. In an acidic (e.g., glacial acetic acid) environment, zein molecules tend to lie flat and the hydrophobic side is in contact with the air due to electrostatic attraction, leading the zein-based film to have relatively lower surface roughness and higher hydrophobicity (Figure 2B). In a neutral (e.g., aqueous ethanol) environment, zein molecules tend to be arranged vertically and the polar AA residues at the top are in contact with the air due to the weak electrostatic repulsion between zein molecules and the carrier, leading the zein-based film to have relatively higher surface roughness and worse hydrophobicity (Figure 2C). However, when acidic solvents are used as food coatings for preservation, those acidic components remain on the products accompanying coatings during storage and decompose and dissolve during cooking or chewing, which may affect the pH value of food systems and even bring undesirable organoleptic properties to the coated foods.

At present, studies on film-forming solvents mainly take the solubility of prolamins as the evaluation index, while there are few studies on the relationship between solvent selection and the properties of the resulting films.

### 2.4. Coating Processes

Common coating methods include dipping, spraying, fluidized-bed, panning/pan-coating, etc. Coating methods are totally different from the packaging method where the film is cast initially and then assembled. The final application effects of coating on foods depend not only on the quality of coating, but also on the properties of coated foods (e.g., shape and size), as well as the nature of the coating and the surface properties (e.g., surface tension, density, and viscosity) [49,50]. Different coating methods have their own advantages and disadvantages (Table 2) and should be selected according to the properties of coated foods and coating solutions as well as the desired purpose in practice [50].

#### 2.4.1. Dipping

**Figure 3:**
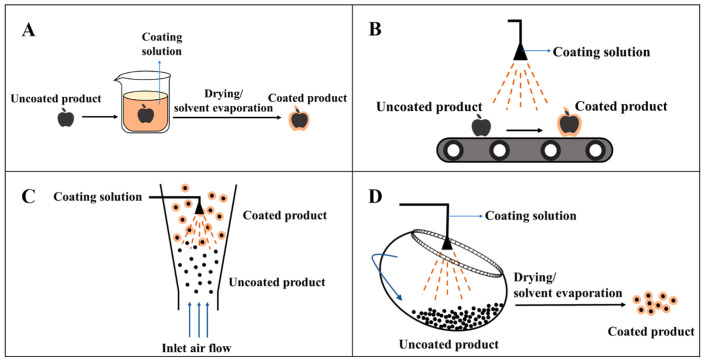
Coating techniques ((**A**): Dipping coating method, (**B**): Spraying coating method, (**C**): Fluidized-bed coating method, (**D**): Pan-coating method) [51,54].

The dipping coating method is currently the main method in laboratory coating application and also the most common method for food coating due to its simple operation and low cost. The dipping method is generally divided into three steps: firstly, foods are immersed into the coating solution for a while to ensure that foods are exposed to a sufficient amount of coating solution and the contacting time between foods and coating solution is long enough; secondly, foods are taken out and left to stand still, and a thin layer of coating is formed on the surface of foods due to surface tension; thirdly, foods are dried at room temperature or under heated conditions, and then a dry film is formed on the surface of foods after evaporating solvents (Figure 3A). The dipping and drying steps can also be cycled alternately if necessary [51]. The dipping method can be used to completely coat the foods with uneven surface or complex shape, but it may cause the dilution of the coating solution. When the adhesion of the coating solution on the surface of foods is poor, multiple soakings may be required to ensure the complete coating of foods, or a complete coating may not even be obtained [52]. Cisneros-Zevallos and Krochta [53] used the dipping method to coat apples and found that the coating thickness of regular objects (flat plates) has a close relationship with the viscosity, density, and solvent evaporation rate of the coating solution, and the relationship is also applicable to irregular objects (apple). However, the coating formed on the surface of a non-horizontal plane or an irregular object by the dipping method has uneven thickness due to the fluidity of coating forming liquid. It is difficult for the dipping method to be applied for massive continuous production in practice.

#### 2.4.2. Spraying

The spraying coating method (Figure 3B) has the advantages of uniform coating, less cross-contamination, and controllable film thickness. Spraying coating can be used for coated foods with large surface area and is also suitable for two or more continuous operations. Therefore, spraying coating is widely used on industrial and commercial scales [49]. However, spraying coating requires the coating solution to have high fluidity and it is generally used for low-viscosity coating solutions. The droplets formed by spraying are influenced by the physical properties of the coating solution, and each material has its own flow behavior. Therefore, studying the rheological behavior of coating liquid is crucial to the selection of atomization pressure and nozzle shape and design [49]. In recent years, studies on combining the electrostatic technique with the spraying technique have been reported [55,56]. Compared with traditional spraying, electrostatic spraying has the advantages of controllable droplet size, uniform distribution, reduced waste, and forming more continuous and uniform coatings [57].

#### 2.4.3. Fluidized-Bed

The fluidized-bed coating method has been the focus of research for various critical applications in the chemical, pharmaceutical, and food industries. It was originally developed for use in the pharmaceutical industry. In the food industry, it is suitable for the coating of dry particles with low density and small size (e.g., wheat [58], peanut [54]). Briefly, this method achieves the purpose of uniform coating by using a fixed nozzle to spray the coating solution onto the surface of fluidized powder/granular products [52]. The particles to be coated are risen from the bottom under the action of airflow and maintain sufficient velocity when passing through the spraying area in order to support the particles to continuously be risen and fluidized (Figure 3C). There are three different spraying methods for fluidized-bed coating: top spraying, bottom spraying, and rotary fluidized-bed, among which the top spraying method is more suitable for batch production and is more common in the food industry. The main disadvantage of fluidized-bed coating is the uncontrolled agglomeration of the coated particles. This is because the coating solution forms liquid bridges among the particles after wetting food particles and these liquid bridges solidify and form agglomerates as the solvent in coating liquid evaporates. However, according to this feature, fluidized-bed coating can be used in production processes aimed at the agglomeration of powder products into particles, which can well reduce dust, improve production safety, and give good dissolution and dispersion capabilities to the product [59]. To ensure product quality, manual real-time observation is required, which leads to a significant increase in production costs [60].

#### 2.4.4. Panning

The pan-coating method is to initially place the products in a large tilting rotating pot, followed by pouring or spraying the coating solution into the pot; with the continuous rotation of the pot, the coating solution is evenly distributed on the surface of products, and then forms the film after drying at room temperature or high temperature (Figure 3D). Pan-coating is more suitable for spherical, ellipsoidal, and quasi-spherical foods. Therefore, this method is commonly used in the pharmaceutical, confectionery and chocolate, and extruded products industries. In the process of coating, moisture and debris in foods will contaminate the coating solution in the pot and solvents volatilize continuously, which leads pan-coating to have a discontinuous operation, long operation time, difficult cleaning, high cost, and high requirements for the scale and shape of the products to be coated.

## 3. Modification of Prolamin-Based Coatings

Single-prolamin-based films usually have high brittleness, low mechanical strengths, and poor stability. To better meet the application requirements, it is often necessary to use various methods to modify and improve the performance of prolamin-based films. Common modification methods include the addition of plasticizers, the compounding of other edible ingredients, enzymatic methods, physical methods (e.g., thermal cross-linking, ultraviolet rays, γ radiation), chemical methods (e.g., formaldehyde vapor, acetylation, succinylation, glycosylation), etc. [61,62,63]. However, the modifications by ultraviolet rays, radiation, or formaldehyde vapor may have adverse impacts on coated foods since prolamin-based films used for coating are directly formed on the surface of foods, and food safety issues such as chemical reagent residues may occur. Thus, these methods should be avoided as much as possible in practical applications.

### 3.1. Mechanical Performance Improvement

Generally, the mechanical properties of films can be expressed by three parameters: tensile strength, elongation at break, and Young’s modulus, which are important indicators for evaluating the tensile load capacity and toughness of films. When protecting coated foods during transportation, storage, and processing, films are required to have strong press-bearing capacity and puncture resistance. In this case, it is very important to improve the tensile strength of films. Cross-linking polymerization can greatly improve the tensile strength of films. Byaruhanga et al. [64] thermally cross-linked the wet kafirin by microwave-heating and found that the maximum tensile strength of the resulting film increases by 39%, the elongation at break decreases by 37%, and the film becomes firmer with reduced plasticity. In recent years, tannin as a natural cross-linking agent has been widely studied in improving the mechanical properties of prolamin-based films [65,66,67,68]. Tannin is a secondary metabolite of plants and has strong complexation with proteins. This is because tannin can interact with cereal proteins through non-covalent bonds (hydrogen bonds and hydrophobic interactions) and thus larger protein macropolymers are formed, reducing the mobility of peptide chains and changing the rheological properties of proteins as well as the mechanical properties of protein-based films [68]. Regardless of thermal cross-linking and chemical cross-linking, the tensile strength of the resulting films is enhanced, basically accompanying the reduced elongation at break (strain). In terms of application effects, coatings prepared through these films are more like “hard shells”, which can provide the mechanical damage protection for coated foods (such as fruits, eggs, medicines, etc.), and provide a hard and crunchy taste when chewing. Compared with polysaccharides-based and lipid-based films, protein-based films have impressive gas barrier properties and better mechanical properties [69]. On some occasions (such as soft candy coating, soluble condiment packaging, etc.), however, films need to provide higher flexibility similar to that of plastic bags. Common synthetic polymers used for plastic bags are low-density and high-density polyethylene films, which have the elongation at break of up to 1000% [70]. Therefore, to meet the demand, it is necessary to add plasticizers into the prolamin-based film-forming solution to increase the elongation at break of the resulting films. The addition of plasticizers can modify the films by reducing the interaction among protein molecule chains and increasing the free volume. The common food plasticizers contain glycerin, fatty acids, propylene glycol, polyethylene glycol, galactose, dibutyl tartrate, sorbitol, sucrose, etc., among which glycerin is the most commonly used [71,72,73]. The structures of different plasticizers determine that they have different plasticizing effects. Pommet et al. [74] pointed out that the plasticizing efficiencies are related to the molar ratios of plasticizers. Wu [75] compared the plasticizing effects of glycerin, oleic acid, and polyethylene glycol on zein-based films and proposed that the plasticizing effects are related to the polarity of both plasticizers and prolamins. Water molecules can penetrate into prolamin-based films after combining with the hydrophilic groups in plasticizers, and then combine with prolamin molecules through hydrogen bonds, producing lubrication. When the plasticizer (such as oleic acid) is less polar, less water molecules are bound, resulting in poor plasticizing effects. When the plasticizer (such as glycerol) is strongly polar, the binding of prolamins with the plasticizer is also weak due to the presence of a large number of hydrophobic groups in prolamins, and the plasticizer precipitates out after prolamin-based films are formed, which also results in a poor plasticizing effect. Only the plasticizers (such as polyethylene glycol) with moderate polarity may have the most effective plasticizing effects. The addition of plasticizers should be taken into consideration comprehensively since it not only affects the mechanical properties but also the barrier properties and water resistance of the resulting films.

### 3.2. Barrier Performance Improvement

Water and oxygen inevitably participate in many degradation and deterioration reactions in foods, such as oxidation reaction, microbial growth, enzymatic browning, etc., resulting in changes in color, odor, taste, and texture. Coating is one of the effective means to alleviate such problems. Therefore, the barrier properties of coatings are crucial in applications. Generally, the barrier properties of coatings are investigated from the following three aspects: gas barrier properties (including barriers to oxygen, carbon dioxide, and water vapor), water barrier properties, and oil barrier properties.

Prolamin-based film is a film with a porous network structure formed by intermolecular interactions. The transport of gas/water/oil molecules in edible films can be divided into three steps: (1) molecules adsorb on the surface of edible films; (2) molecules diffuse from one side of edible films to the other; and (3) molecules desorb from the surface of edible films [76]. Therefore, the existing modification methods for improving the barrier properties of edible films can be roughly divided into two categories based on the principle: one is to strengthen the intermolecular interaction force and the network structures of edible films through cross-linking; the other is to add or compound insoluble particles or small molecule particles into the edible film matrix to create a more tortuous diffusion path, thereby prolonging the migration time of gas/water/oil molecules. Prolamin-based films themselves exhibit superior gas barrier properties compared to lipid-based and polysaccharide-based films and even synthetic polymer films [77]. Therefore, there are few studies on the modification of prolamin-based films for the purpose of improving gas barrier properties. In addition to the cross-linking reaction, complexing lipids with prolamins is a common method to improve the water and water vapor barrier properties of prolamin-based films. Lu et al. [78] found that a low addition (0.3 g/g) of monoglycerides can improve the water vapor and oil barrier properties of zein-based films; however, when an excessive amount of monoglycerides is used, the interaction between monoglycerides and zein molecules weakens the interaction force among zein macromolecules, and the surface of the resulting films is uneven, increasing the water vapor transmission rate. Masamba et al. [79] reported that transglutaminase cross-linking improves the mechanical properties and water barrier properties of composite zein-based films. In addition, they found that drying temperature and pH have significant effects on the mechanical properties and water barrier properties of the resulting films. Notably, the addition of plasticizers can lower the glass transition temperature of films and increase the permeability of films. Masamba et al. [80] compared the plasticizing effects of glycerin, sorbitol, ethylene glycol, and propylene glycol on the zein-oleic acid composite films and proposed that: (1) the gas permeability of films is related to the molecular weight of plasticizers, and high-molecular-weight plasticizer increases the air permeability of films more obviously than low-molecular-weight plasticizer; (2) the differences in the hydrophilicity of plasticizers also affect the water vapor permeability of films, and hydrophilic plasticizer increases the water permeability of films. All barrier properties of edible films are greatly influenced by film composition and environmental conditions (e.g., relative humidity and temperature). The permeability of prolamin-based films is very sensitive to the relative humidity of the environment, and oxygen permeability increases significantly under higher relative humidity conditions. Therefore, it is very important to keep the environment dry during food preservation.

### 3.3. Water Resistance Improvement

**Figure 4:**
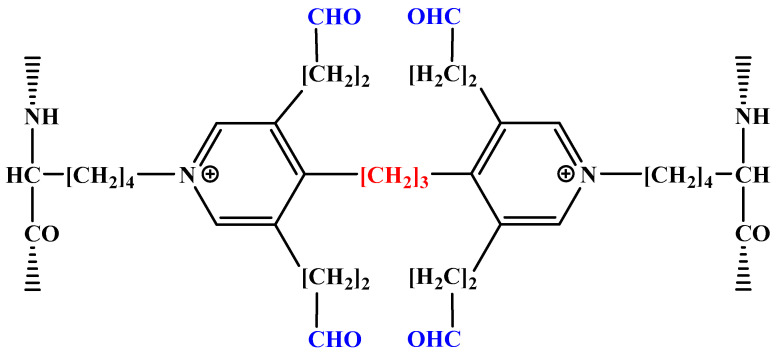
Schematic structure of cross-linking reaction between glutaraldehyde and protein.

In the food industry, some processing and storage processes often need to be carried out under high water activity conditions, such as soaking, pickling, cooking, canned packaging, etc. The barrier ability of prolamin-based films will be greatly reduced or even destroyed when contacting water or staying under wet conditions for a long time. Therefore, the poor stability of prolamin-based films in water limits its application in high water activity environments. At present, improving the intermolecular cross-linking cohesion of proteins is a common method to improve the water stability of biomaterials such as prolamin-based films and fibers. The reported cross-linking agents include citric acid, oxidized sucrose, olive leaf extract, cysteine and aldehydes, etc. [81,82,83]. Among them, aldehydes are considered to be the most effective cross-linking agents. For example, the traditional cross-linking agent glutaraldehyde can cross-link collagen [84], wheat gluten [85], gliadin [86], zein [87], kafirin [88], etc., by forming a stable ring structure between the free amino group of peptide chains and the carbonyl group of aldehydes, greatly improving the water stability of the resulting films. Figure 4 shows the schematic structure of glutaraldehyde cross-linking with protein. Although glutaraldehyde can significantly improve the performance of protein-based films in all aspects and is widely known for its low cost, its residual cytotoxicity has been attracting more and more attention [89]. Therefore, safer modification methods are actively being sought to replace glutaraldehyde cross-linking, such as transglutaminase cross-linking.

**Figure 5:**
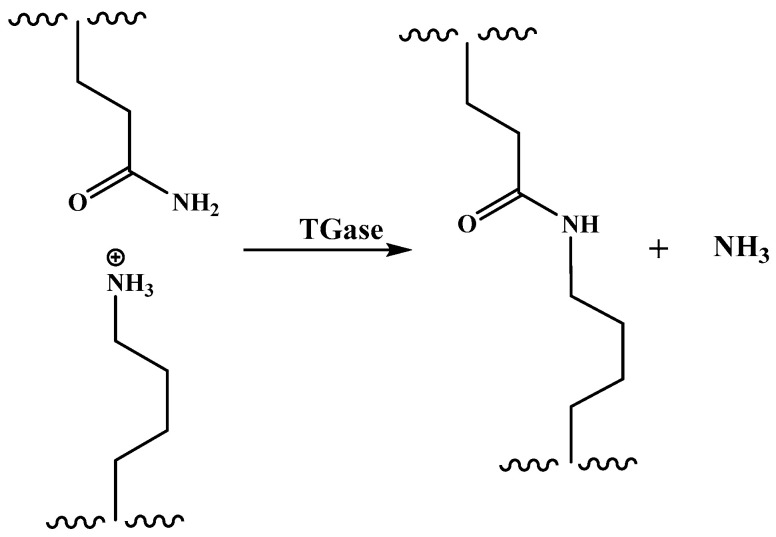
Schematic structure of cross-linking reaction between transglutaminase and protein.

**Figure 6:**
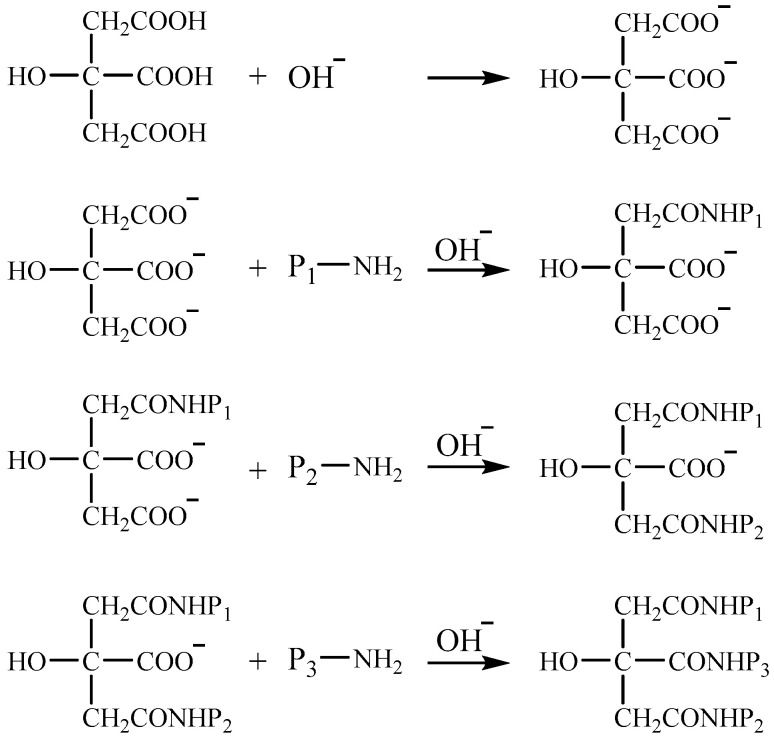
Schematic structure of the cross-linking reaction between citric acid and protein.

Transglutaminase is a natural protein cross-linking agent, mainly through the acyl transfer reaction between the γ-carboxamide group of glutamine (acyl donor) and the ε-amino group of lysine residues (acyl acceptor) to form intramolecular and intermolecular cross-linking of ε-(γ-glutamyl)lysine (Figure 5). Some researchers believe that prolamins cannot provide the acyl acceptors for cross-linking reactions and are unsatisfactory substrates for transglutaminase reactions due to the low content of lysine. Anyango et al. [88] investigated the kafirin microparticle films before and after adding transglutaminase and found that the micronization of kafirin can improve the water stability of the resulting films to a certain extent, but the water stability of films with adding transglutaminase becomes worse. They believed that it may be because the transglutaminase-induced deamidation with insufficient lysine results in poor compatibility of kafirin microparticles and inhomogeneous films. Reddy and Yang [90] used high-concentration urea instead of aqueous ethanol as the solvent to dissolve gliadin, and found that urea converts intramolecular disulfide bonds into intermolecular disulfide bonds, thereby increasing the molecular weight of gliadin and successfully improving the water stability of gliadin-based films without additional cross-linking agents. Wang et al. [91] co-dispersed sodium caseinate and zein in 60% aqueous ethanol, and cross-linked with citric acid to form composite films. The hydrophobicity and water stability of the composite films both improve due to the formation of compact structures by the interaction between sodium caseinate and zein. Reddy et al. [85] investigated the effect of different conditions on the elongation at break and tensile strength in wet cross-linking of gliadin films with citric acid and suggested that: higher alkaline pH conditions may be more favorable for the cross-linking reaction to proceed. A schematic diagram of the cross-linking reaction between citric acid and protein (Figure 6) is proposed [92].

In addition to using cross-linking agents to chemically or biologically cross-link prolamins, blending prolamins with water-resistant materials is also a feasible method to improve the water resistance of films with the priority of ensuring their edibility. Lu et al. [93] prepared water-stable zein/ethyl cellulose composite nanofibers with good mechanical properties by blending and spinning the hydrophobic, physiologically inert cellulose derivative ethyl cellulose and zein. The nanofiber film prepared with this composite fiber can maintain the original appearance and the basically unchanged water contact angle (the hydrophobic property) after 56 h of immersion in buffer solution. The compounding of materials may cause uneven compatibility and increased cost. Therefore, seeking non-toxic, green, safe, economical, and easy-to-operate methods is still the focus of current research to improve the performance of prolamin-based films.

In addition to the abovementioned commonly used edible film modification methods, some additional methods of prolamin-based film modification such as nanoparticle compounding, electron beam irradiation, cross-linking via the Melad reaction, etc., may affect the different properties and application effects of the films (Table 3).

### 3.4. Other Performance Improvement

In addition to the requirements for the physical properties of coatings themselves, some additional properties are also expected in some food applications, such as antibacterial properties, anti-oxidation, nutritional enhancement or better gloss, and more pleasant smell. Coatings can also help bioactive substances in coatings slowly and continuously release to the surface of foods, thus maintaining the uniform distribution of bioactive substances and the stability of their antibacterial and antioxidative properties during storage [99]. The antibacterial additives and the applications of antibacterial prolamin-based film and coating are listed in Table 4.

Most essential oils have good antibacterial and antioxidative effects, such as mustard essential oil, cinnamon essential oil, thyme essential oil, oregano essential oil, rosemary essential oil, and olive oil. They have the advantages of biodegradability, non-toxicity, controllable release, and high activity, and thus are often used as the additives in edible films to enhance antibacterial properties. Phenolic compounds present in essential oils disrupt the permeability of bacterial membranes and thus hinder the enzymatic activity of microorganisms. Kashiri et al. [100] reported that thyme essential oil can effectively improve the antibacterial properties of zein-based films and carvacrol and thymol are the main antibacterial active substances in thyme essential oil. They exhibit a variety of antibacterial activity modes: firstly, they cause the expansion and structural instability of bacterial membranes, thereby increasing the leakage of cytoplasm; secondly, carvacrol can be used as a proton exchanger to reduce the power of protons on the plasma membrane due to its weak acidic nature. In addition, as natural antioxidants, phenolic compounds have strong hydrogen donating capacity, which can reduce the activity of free radicals or completely eliminate free radicals during oil oxidation, thereby inhibiting the oxidation reaction [101]. Adding metal or metal oxide nanoparticles (such as Ag, ZnO, and CuO) into packaging films is another effective option to improve antimicrobial properties. Metal nanoparticles slowly release dissociated ions, which interact with the negative charges on bacterial cell walls, resulting in cell death [102]. Regarding the edible safety of nanomaterials, although some in vitro and in vivo studies have been carried out, the conclusions drawn are still contradictory and controversial [103]. Therefore, a cautious approach should be taken in the application of nanomaterials in food coating.

**Table 4:** Antibacterial and antioxidant applications of prolamin.

Prolamin	Additive	Apply to	Application	Additional Effect	Ref.
Zein	Eugenol + Carvacrol + Thymol	Inhibits *L. innocua* and *E. coli*	Whole melons	—	[104]
	Nisin + ethylenediaminetetraacetic acid	Inhibits *E. coli*, *E. aerogenes* and *C. freundii*	Commercial fish balls	—	[105]
	gallic acid	Inhibits *C. jejuni*	—	—	[106]
	ethyl-Nα-dodecanoyl-L-arginate hydrochloride (LAE)	Inhibits *L. monocytogenes* and *E. coli*	—	LAE addition (Addition amount: 5%, 10%) does not cause substantial changes in morphological, optical, thermal, mechanical and barrier properties.	[107]
	Cinnamon essential oil + Chitosan nanoparticles	Inhibits *E. coli* and *S. aureus*	—	The tensile strength of the film increases, and the elongation at break decreases.	[108]
Kafirin	Citral	Inhibits bacterial growth	Fresh chicken fillets	The maximum stress and stiffness of the film decrease, while the fracture strain and yield stress increase	[109]
	Quercetin	Delays lipid oxidation	Fresh chicken fillets	—	
Gliadin	Cinnamaldehyde	Inhibits *E. coli* and *S. aureus*	—	The chemical cross-linking of cinnamaldehyde and protein is carried out under alkaline conditions, and the mechanical properties and water stability of the film improve after cross-linking	[110]
	Cinnamaldehyde	Inhibits *P. expansum* and *A. niger*	Bread and cheese spread	—	[111]
	Zataria multiflora Boiss essential oil	Inhibits *B. subtilis* and *L. monocytogenes*, and has antioxidant properties	Smoked salmon fish fillet	—	[112]

## 4. Conclusions and Further Remarks

The concept of sustainable development is deepening, and the transition from plastic packaging to biological packaging is proceeding rapidly. In the long run, biological packaging materials will play an increasing role in our daily life because of their convenience, effectiveness, greenness, and safety. At present, studies on prolamin-based films have obtained certain achievements. However, there are still some issues that need to be studied more deeply in the future.

(1) Except for commercial zein, other prolamins still face low extracting yield, long extracting time, and high cost, which limits their development in actual production and application. The extracting processes using industrial by-products as the raw materials should be optimized and strengthened to improve the comprehensive utilization efficiency of grains. At the same time, attention should be paid to the recovery and reuse of extracting solvents, so as to reduce the pollution and production costs;
(2) Most studies on the extracting processes of prolamins mainly take the extracting yield as the evaluation index. In fact, different extracting methods, extracting solvents, and extracting conditions largely impact the structures and composition of the final prolamin product, ultimately affecting the performance and application effect of the resulting films. Various means should be used to characterize the properties of obtained prolamins, such as the secondary, tertiary, and quaternary structures of prolamins, AA composition, and disulfide bond content, etc., in order to clarify the relationship between the extracting processes and the properties of the resulting films;
(3) The studies on prolamin-based films mainly focus on improving their performance by optimization of the film-forming processes or modifications. However, when prolamin-based films are actually used for food coating, they are very likely to encounter problems such as irregular food shape, poor adhesion between the food surface and the coating solution, and unsatisfactory application environment. Thus, the physical properties of the coating solution (such as rheology, viscosity, density, surface tension, etc.) should be studied and adjusted from the perspective of application, so as to find out the composition and properties of the coating solution suitable for the coating processes;
(4) Although prolamins have good water insolubility, prolamin-based films swell under the environment of high-water activity, which makes the barrier performance of the film plummet. It is difficult to provide continuous barrier protection for coated foods. At present, the more effective modification method is to greatly increase the cross-linking degree of prolamin-based films through chemical cross-linking or cross-linking such as irradiation. However, these modification methods are difficult to implement in food coating applications. Moreover, prolamin-based films modified by such methods usually run counter to their original advantages of being edible and easy to degrade. Therefore, seeking non-toxic, green, safe, economical, and easy-to-operate methods should be the research focus to improve the stability of coatings;
(5) Although prolamin-based films do not have a waxy texture like lipid-based films, some prolamins themselves carry a special odor, which may be unpleasant to some users and consumers, and which can be masked or eliminated by some processing means in the subsequent research. And for food coating, it is worth noting how to balance the mechanical properties of the coatings and the resistance of the coatings during chewing.

## Figures and Tables

**Table 1:** Amino acid composition of corn, sorghum, wheat, and barley prolamin [23].

Amino Acid	Zein	Kafirin	Gliadin	Hordein
Ala	11.3	12.4	2.3	1.6
Arg	1.1	1.0	2.4	1.6
Asp	3.9	6.5	1.3	0.7
Cys	1.9	0.4	2.7	0.5
Glu	20.7	30.0	38.4	42.0
Gly	3.8	1.1	3.3	1.7
His	2.1	0.9	1.6	0.2
Ile	3.7	4.8	3.7	5.8
Leu	18.7	19.2	7.0	6.0
Lys	0.2	0.1	0.5	5.4
Met	1.9	1.0	0.6	0.8
Phe	5.3	6.4	4.7	2.3
Pro	8.7	10.0	15.0	22.7
Ser	5.9	4.1	7.7	2.1
Thr	3.1	2.6	2.8	2.6
Tyr	3.2	5.5	1.5	2.3
Val	4.5	5.0	4.7	1.9

**Table 2:** Principle, advantages, and disadvantages of coating techniques.

Method	Principle	Advantages	Disadvantages
Dipping	The uncoated product is dipped into the film-forming liquid; the solvent in the coating-forming liquid on the surface of the product volatilizes to form a coating	Simple operation and low cost; can be applied to irregular surfaces	Uneven coating thickness; cannot be serialized
Spraying	The uncoated product is conveyed by a conveyor belt and passes under a fixed coating solution nozzle, where the coating solution is sprayed on the product and then cured into a coating	Uniform coating, less cross-contamination, and controllable coating thickness; can be used for coated foods with large surface area	Requires the coating solution to have high fluidity and is generally used for low-viscosity coating solutions
Fluidized-bed	The particles to be coated are risen from the bottom under the action of airflow, followed by using a fixed nozzle to spray the coating solution onto the surface of fluidized powder/granular products	Suitable for batch production; can be used for dry particles with low density and small size	Uncontrolled agglomeration of the coated particles
Panning	The uncoated products are placed into a rotating pan; as the pan rotates, the coating solution is sprayed out and adheres onto the surface of the product	Can produce products in large quantities at the same time	Discontinuous operation, long operation time, difficult cleaning, high cost, and high requirements for the scale and shape of the products to be coated

**Table 3:** Modification and application effects of prolamin.

Prolamin	Modification	Modification Effect	Application	Application Effects	Ref.
Zein	Zein + sunflower oil complex	Large decrease in tensile Young’s modulus and strength with increasing level of sunflower oil in zein films	Wheat bread	Coated breads exhibited retardation in moisture migration from crumb to crust compared to uncoated counterparts	[94]
Zein	Zein + ε-polylysine nanoparticles complex	—	Avocado	By day 36 in ambient storage in this study, coated avocados retained enough of their initial physical appearance and texture	[95]
Kafirin	Electron beam irradiate kafirin-quercetin film	Irradiation significantly increased mechanical and thermal properties of KQ films, while decreasing water vapor permeability, water solubility, and transparency	Cod fillets during cold storage at 4 °C.	Shelf life of cod fillets wrapped in irradiated prolamin film increased from 4 to 7 days compared to fillets prepared with polyethylene coating	[96]
Kafirin	TEMPO-oxidized cellulose nanofiber + kafirin cross-linked by Maillard reaction	Young’s Modulus shows significant increases at 0.5% of TO-CNF; with a gradual decrease at 3% of TO-CNF	—	—	[97]
Gliadin	Dialdehyde polysaccharides + citric acid cross-linked	The mechanical properties, water-resistant properties, thermal stability, antibacterial properties of the gliadin films were all advanced	—	—	[98]

## Data Availability

Data will be made available on request.

## References

[1] Gaikwad K.K., Singh S., Negi Y.S. (2020). Ethylene scavengers for active packaging of fresh food produce. Environ. Chem. Lett..

[2] Shao L., Chen S., Wang H., Zhang J., Xu X., Wang H. (2021). Advances in understanding the predominance, phenotypes, and mechanisms of bacteria related to meat spoilage. Trends Food Sci. Technol..

[3] Fernández-Pan I., Carrión-Granda X., Maté J.I. (2014). Antimicrobial efficiency of edible coatings on the preservation of chicken breast fillets. Food Control.

[4] Han J.W., Ruiz Garcia L., Qian J.P., Yang X.T. (2018). Food packaging: A comprehensive review and future trends. Compr. Rev. Food Sci. Food Saf..

[5] Chen Z.H., Chen J.Q., Liu X.X., Wang X. (2022). Research progress of application of polysaccharides, proteins and their composite coatings in preservation of postharvest berries. Storage Process.

[6] Ma C.F. (2017). The Effect of Modified Atmosphere Packaging and Coating Process on the Quality of Frozen Tilapia Fillets. Master’s Thesis.

[7] Tran P.H., Duan W., Lee B., Tran T.T. (2019). The use of zein in the controlled release of poorly water-soluble drugs. Int. J. Pharm..

[8] Fan Y.Z., Lu R.X., Zhu H.Y., Miao M. (2023). Fresh-keeping effect of pullulan polysaccharide coating treatment on cherry tomato. Food Ferment. Ind..

[9] Zhang Q., Yin L.J., Chen F.S. (2020). Research progress on polysaccharide-based edible film. Cereals Oils.

[10] Friedman M., Juneja V.K. (2010). Review of antimicrobial and antioxidative activities of chitosans in food. J. Food Prot..

[11] Cheng K., Demirci A., Catchmark J.M. (2011). Pullulan: Biosynthesis, production, and applications. Appl. Microbiol. Biotechnol..

[12] Rodrigues D.C., Cunha A.P., Brito E.S., Azeredo H.M., Gallao M.I. (2016). Mesquite seed gum and palm fruit oil emulsion edible films: Influence of oil content and sonication. Food Hydrocoll..

[13] Wang R.L., Bian K., Xu S.Y. (2002). Study on preventing fat deterioration in nuts coated with edible film from vegetable proteins. J. Henan Univ. Technol. (Nat. Sci. Ed.).

[14] Hassan B., Chatha S.A.S., Hussain A.I., Zia K.M., Akhtar N. (2018). Recent advances on polysaccharides, lipids and protein based edible films and coatings: A review. Int. J. Biol. Macromol..

[15] Shewry P.R. (2002). The major seed storage proteins of spelt wheat, sorghum, millets and pseudocereals. Pseudocereals and Less Common Cereals.

[16] Chen W.Y., Mu Y., Zhu A.F., Zhang B.H. (2022). Evaluation of the preparation and preservation effects of zein film. Modern Food Sci. Technol..

[17] Hager J.V., Rawles S.D., Xiong Y.L., Newman M.C., Webster C.D. (2019). Edible corn-zein-based coating incorporated with nisin or lemongrass essential oil inhibits listeria monocytogenes on cultured hybrid striped bass, morone chrysops× morone saxatilis, fillets during refrigerated and frozen storage. J. World Aquacult. Soc..

[18] Zhang C.H., Chang N., Gao H.N. (2010). Preparation of edible composite film with wheat gluten and zein and its application in hazelnut kernels preservation. China Oils Fats.

[19] Lu L.L. (2020). Preparation and Functional Study of Novel Zein Drug Carriers. Master’s Thesis.

[20] Versino F., Lopez O.V., Garcia M.A., Zaritzky N.E. (2016). Starch-based films and food coatings: An overview. Starch-Stärke.

[21] Zang M., Shan C.Y., Ma Y.F., Nei X., Ma S.H. (2017). Research progress of cereal gliadin. Chin. Wild Plant Resour..

[22] Derose R.T., Ma D., Kwon I., Hasnain S.E., Klassy R.C., Hall T.C. (1989). Characterization of the kafirin gene family from sorghum reveals extensive homology with zein from maize. Plant Mol. Biol..

[23] Cao W., Li F., Huang Q.R. (2015). The extraction of hordein and properties of hordein. Grain Process..

[24] Xiao J., Li Y., Li J., Gonzalez A.P., Xia Q., Huang Q. (2015). Structure, morphology, and assembly behavior of kafirin. J. Agric. Food. Chem..

[25] Taylor J., Taylor J., Dutton M.F., De Kock S. (2005). Identification of kafirin film casting solvents. Food Chem..

[26] Qazanfarzadeh Z., Kadivar M., Shekarchizadeh H., Porta R. (2021). Rye secalin characterisation and use to improve zein-based film performance. Int. J. Food Sci. Technol..

[27] Xu J., Li Y. (2023). Wheat gluten–based coatings and films: Preparation, properties, and applications. J. Food Sci..

[28] Hernández-Muñoz P., Lagarón J.M., López-Rubio A., Gavara R. (2004). Gliadins polymerized with cysteine: Effects on the physical and water barrier properties of derived films. Biomacromolecules.

[29] Hernández-Muñoz P., Kanavouras A., Ng P.K., Gavara R. (2003). Development and characterization of biodegradable films made from wheat gluten protein fractions. J. Agric. Food. Chem..

[30] Oliviero M., Di Maio E., Iannace S. (2010). Effect of molecular structure on film blowing ability of thermoplastic zein. J. Appl. Polym. Sci..

[31] Belton P.S., Delgadillo I., Halford N.G., Shewry P.R. (2006). Kafirin structure and functionality. J. Cereal Sci..

[32] Dong S., Xu H., Tan J., Xie M., Yu G. (2017). The structure and amphipathy characteristics of modified γ-zeins by sds or alkali in conjunction with heating treatment. Food Chem..

[33] Holding D.R. (2014). Recent advances in the study of prolamin storage protein organization and function. Front. Plant Sci..

[34] Shukla R., Cheryan M. (2001). Zein: The industrial protein from corn. Ind. Crop. Prod..

[35] Hou M.Y., Fan W., Xu Y. (2020). Extraction and characterization comparison of prolamin from wet and dried distiller’s grains of baijiu. Food Ferment. Ind..

[36] Wang Y., Tilley M., Bean S., Sun X.S., Wang D. (2009). Comparison of methods for extracting kafirin proteins from sorghum distillers dried grains with solubles. J. Agric. Food. Chem..

[37] Selling G.W., Woods K.K. (2008). Improved isolation of zein from corn gluten meal using acetic acid and isolate characterization as solvent. Cereal Chem..

[38] Parris N., Dickey L.C. (2001). Extraction and solubility characteristics of zein proteins from dry-milled corn. J. Agric. Food. Chem..

[39] Schober T.J., Bean S.R., Tilley M., Smith B.M., Ioerger B.P. (2011). Impact of different isolation procedures on the functionality of zein and kafirin. J. Cereal Sci..

[40] Anderson T.J., Lamsal B.P. (2011). Development of new method for extraction of α-zein from corn gluten meal using different solvents. Cereal Chem..

[41] Lai C.J., Wu L.Y., Hu L.F., Tu J., Dong W.H. (2021). Aggregation state and structural properties of zein in different solvents. Mod. Food Sci. Technol..

[42] Dianda N., Rouf T.B., Bonilla J.C. (2019). Effect of solvent polarity on the secondary structure, surface and mechanical properties of biodegradable kafirin films. J. Cereal Sci..

[43] Pena-Serna C., Lopes-Filho J.F. (2013). Influence of ethanol and glycerol concentration over functional and structural properties of zein–oleic acid films. Mater. Chem. Phys..

[44] Kim S., Xu J. (2008). Aggregate formation of zein and its structural inversion in aqueous ethanol. J. Cereal Sci..

[45] Yin Y., Yin S., Yang X., Tang C., Wen S., Chen Z., Xiao B., Wu L. (2014). Surface modification of sodium caseinate films by zein coatings. Food Hydrocoll..

[46] Chen H., Wang J., Cheng Y., Wang C., Liu H., Bian H., Pan Y., Sun J., Han W. (2019). Application of protein-based films and coatings for food packaging: A review. Polymers.

[47] Matsushima N., Danno G., Takezawa H., Izumi Y. (1997). Three-dimensional structure of maize α-zein proteins studied by small-angle X-ray scattering. Biochim. Et Biophys. Acta (BBA)-Protein Struct. Mol. Enzymol..

[48] Shi K., Kokini J.L., Huang Q. (2009). Engineering zein films with controlled surface morphology and hydrophilicity. J. Agric. Food. Chem..

[49] Andrade R.D., Skurtys O., Osorio F.A. (2012). Atomizing spray systems for application of edible coatings. Compr. Rev. Food. Sci. Food Saf..

[50] Boddula R., Ahamed M.I., Asiri A.M. (2020). Polymers Coatings: Technology and Applications.

[51] Luciano C.G., Caicedo Chacon W.D., Valencia G.A. (2022). Starch-based coatings for food preservation: A review. Starch-Stärke.

[52] Atieno L., Owino W., Ateka E.M., Ambuko J. (2019). Influence of coating application methods on the postharvest quality of cassava. Int. J. Food Sci..

[53] Cisneros Zevallos L., Krochta J.M. (2003). Dependence of coating thickness on viscosity of coating solution applied to fruits and vegetables by dipping method. J. Food Sci..

[54] Lin S.Y., Krochta J.M. (2006). Fluidized-bed system for whey protein film coating of peanuts. J. Food Process Eng..

[55] Patel M.K. (2016). Technological improvements in electrostatic spraying and its impact to agriculture during the last decade and future research perspectives—A review. Eng. Agric. Environ. Food.

[56] Sasaki R.S., Teixeira M.M., Fernandes H.C., Monteiro P.M.D.B., Rodrigues D.E., Alvarenga C.B.D. (2013). Parameters of electrostatic spraying and its influence on the application efficiency. Rev. Ceres.

[57] Peretto G., Du W., Avena-Bustillos R.J., De Berrios J., Sambo P., Mchugh T.H. (2017). Electrostatic and conventional spraying of alginate-based edible coating with natural antimicrobials for preserving fresh strawberry quality. Food Bioprocess Technol..

[58] Solís-Morales D., Sáenz-Hernández C.M., Ortega-Rivas E. (2009). Attrition reduction and quality improvement of coated puffed wheat by fluidised bed technology. J. Food Eng..

[59] Nascimento R.F., Ávila M.F., Taranto O.P., Kurozawa L.E. (2022). Agglomeration in fluidized bed: Bibliometric analysis, a review, and future perspectives. Powder Technol..

[60] Prata A.S., Maudhuit A., Boillereaux L., Poncelet D. (2012). Development of a control system to anticipate agglomeration in fluidised bed coating. Powder Technol..

[61] Bisharat L., Barker S.A., Narbad A., Craig D.Q. (2019). In vitro drug release from acetylated high amylose starch-zein films for oral colon-specific drug delivery. Int. J. Pharm..

[62] Li P., Zhang H.J., Guo H., Song J.Y., He X.H., Xu X.H., Zao H., Xin D.H. (2021). Performance of zein film modified by glycosylation and in vitro release analysis of hard capsule. Trans. Chin. Soc. Agric. Eng..

[63] Wang Q., Chen W., Ma C., Chen S., Liu X., Liu F. (2022). Enzymatic synthesis of sodium caseinate-egcg-carboxymethyl chitosan ternary film: Structure, physical properties, antioxidant and antibacterial properties. Int. J. Biol. Macromol..

[64] Byaruhanga Y.B., Erasmus C., Taylor J.R. (2005). Effect of microwave heating of kafirin on the functional properties of kafirin films. Cereal Chem..

[65] Byaruhanga Y.B., Emmambux M.N., Belton P.S., Wellner N., Ng K.G., Taylor J. (2006). Alteration of kafirin and kafirin film structure by heating with microwave energy and tannin complexation. J. Agric. Food. Chem..

[66] Cano A., Andres M., Chiralt A., González-Martinez C. (2020). Use of tannins to enhance the functional properties of protein based films. Food Hydrocoll..

[67] Emmambux M.N., Stading M., Taylor J. (2004). Sorghum kafirin film property modification with hydrolysable and condensed tannins. J. Cereal Sci..

[68] Girard A.L., Teferra T., Awika J.M. (2019). Effects of condensed vs. hydrolysable tannins on gluten film strength and stability. Food Hydrocoll..

[69] Pérez-Gago M.B., Krochta J.M. (2011). Protein-based films and coatings. Edible Coatings and Films to Improve Food Quality.

[70] Sperling L.H. (2005). Introduction to Physical Polymer Science.

[71] álvarez-Castillo E., Ramos M., Bengoechea C., Martínez I., Romero A. (2020). Effect of blend mixing and formulation on thermophysical properties of gluten-based plastics. J. Cereal Sci..

[72] Ghanbarzadeh B., Oromiehie A.R., Musavi M., D-Jomeh Z.E., Rad E.R., Milani J. (2006). Effect of plasticizing sugars on rheological and thermal properties of zein resins and mechanical properties of zein films. Food Res. Int..

[73] Wang X., Wei Q., Wang Y.Y., Yan X., Guo X.F. (2018). Selection and optimization of plasticizers and their effects on moisture barrier property of zein film. J. Henan Univ. Technol. (Nat. Sci. Ed.).

[74] Pommet M., Redl A., Guilbert S., Morel M. (2005). Intrinsic influence of various plasticizers on functional properties and reactivity of wheat gluten thermoplastic materials. J. Cereal Sci..

[75] Wu L.Y. (2010). Study on Modification, Surface Properties and Film Forming Properties of Zein. Ph.D. Dissertation.

[76] Skurtys O., Acevedo C. (2010). Food Hydrocolloid Edible Films and Coatings.

[77] Han J.H. (2014). Edible films and coatings: A review. Innovations in Food Packaging.

[78] Lu Y.N., Cui H.P., Ren H.W., Guo X.F. (2012). Effect of plasticizers on the barrier property of zein film. Cereals Oils.

[79] Masamba K., Li Y., Hategekimana J., Ma J., Zhong F. (2016). Effect of drying temperature and ph alteration on mechanical and water barrier properties of transglutaminase cross linked zein–oleic acid composite films. LWT-Food Sci. Technol..

[80] Masamba K., Li Y., Hategekimana J., Liu F., Ma J., Zhong F. (2016). Effect of type of plasticizers on mechanical and water barrier properties of transglutaminase cross-linked zein–oleic acid composite films. Int. J. Food Eng..

[81] Erdogan I., Demir M., Bayraktar O. (2015). Olive leaf extract as a crosslinking agent for the preparation of electrospun zein fibers. J. Appl. Polym. Sci..

[82] Jiang Q., Yang Y. (2011). Water-stable electrospun zein fibers for potential drug delivery. J. Biomater. Sci. Polym. Ed..

[83] Xu H., Liu P., Mi X., Xu L., Yang Y. (2015). Potent and regularizable crosslinking of ultrafine fibrous protein scaffolds for tissue engineering using a cytocompatible disaccharide derivative. J. Mat. Chem. B.

[84] Xu J.L. (2021). Improving Strategies and Related Mechanisms for Mechanical Properties of Collagen Fiber Edible Film. Ph.D. Dissertation.

[85] Reddy N., Tan Y., Li Y., Yang Y. (2008). Effect of glutaraldehyde crosslinking conditions on the strength and water stability of wheat gluten fibers. Macromol. Mater. Eng..

[86] Prata A.S., Zanin M.H., Ré M.I., Grosso C.R. (2008). Release properties of chemical and enzymatic crosslinked gelatin-gum arabic microparticles containing a fluorescent probe plus vetiver essential oil. Colloids Surf. B Biointerfaces.

[87] Sessa D.J., Mohamed A., Byars J.A., Hamaker S., Selling G.W. (2010). Properties of films from corn zein reacted with glutaraldehyde. J. Appl. Polym. Ence.

[88] Anyango J.O., Taylor J., Taylor J.R. (2011). Improvement in water stability and other related functional properties of thin cast kafirin protein films. J. Agric. Food. Chem..

[89] Casali D.M., Yost M.J., Matthews M.A. (2018). Eliminating glutaraldehyde from crosslinked collagen films using supercritical CO_2_. J. Biomed. Mater. Res. Part A.

[90] Reddy N., Yang Y. (2010). Developing water stable gliadin films without using crosslinking agents. J. Polym. Environ..

[91] Wang S., Chen H., Tong Y., Li Y., Zhang J., Chen C., Ren F., Hou C., Wang P. (2023). Composite films with properties improved by increasing the compatibility of sodium caseinate and zein in a heated 60% ethanol solvent. Food Hydrocoll..

[92] Xu H., Shen L., Xu L., Yang Y. (2015). Low-temperature crosslinking of proteins using non-toxic citric acid in neutral aqueous medium: Mechanism and kinetic study. Ind. Crop. Prod..

[93] Lu H., Wang Q., Li G., Qiu Y., Wei Q. (2017). Electrospun water-stable zein/ethyl cellulose composite nanofiber and its drug release properties. Mater. Sci. Eng. C.

[94] Arancibia M.Y., López-Caballero M.E., Gómez-Guillén M.C., Montero P. (2014). Release of volatile compounds and biodegradability of active soy protein lignin blend films with added citronella essential oil. Food Control.

[95] Mouzakitis C., Sereti V., Matsakidou A., Kotsiou K., Biliaderis C.G., Lazaridou A. (2022). Physicochemical properties of zein-based edible films and coatings for extending wheat bread shelf life. Food Hydrocoll..

[96] Garcia F., Lin W., Mellano V., Davidov-Pardo G. (2022). Effect of biopolymer coatings made of zein nanoparticles and ε-polylysine as postharvest treatments on the shelf-life of avocados (persea americana mill. Cv. Hass). J. Agric. Food Res..

[97] Huang T., Lin J., Fang Z., Yu W., Li Z., Xu D. (2020). Preparation and characterization of irradiated kafirin-quercetin film for packaging cod (gadus morhua) during cold storage at 4 °C. Food Bioproc. Technol..

[98] Lal S.S.S.T. (2019). Tempo-oxidized cellulose nanofiber/kafirin protein thin film crosslinked by maill. Cellulose.

[99] Jia F., Huang Y., Zhao J., Luo S., Hou Y., Hu S.-Q. (2021). Physicochemical and functional properties of dialdehyde polysaccharides crosslinked gliadin film cooperated by citric acid. J. Cereal Sci..

[100] Kashiri M., Cerisuelo J.P., Domínguez I., López-Carballo G., Muriel-Gallet V., Gavara R., Hernández-Muñoz P. (2017). Zein films and coatings as carriers and release systems of zataria multiflora boiss. Essential oil for antimicrobial food packaging. Food Hydrocoll..

[101] Ge X., Huang X., Zhou L., Wang Y. (2022). Essential oil-loaded antimicrobial and antioxidant zein/poly (lactic acid) film as active food packaging. Food Packag. Shelf Life.

[102] Lee J.H., Jeong D., Kanmani P. (2019). Study on physical and mechanical properties of the biopolymer/silver based active nanocomposite films with antimicrobial activity. Carbohydr. Polym..

[103] Becaro A.A., Siqueira M.C., Puti F.C., de Moura M.R., Correa D.S., Marconcini J.M., Mattoso L.H., Ferreira M.D. (2017). Cytotoxic and genotoxic effects of silver nanoparticle/carboxymethyl cellulose on allium cepa. Environ. Monit. Assess..

[104] Boyacı D., Iorio G., Sozbilen G.S., Alkan D., Trabattoni S., Pucillo F., Farris S., Yemenicioğlu A. (2019). Development of flexible antimicrobial zein coatings with essential oils for the inhibition of critical pathogens on the surface of whole fruits: Test of coatings on inoculated melons. Food Packag. Shelf Life.

[105] Lin L.S., Wang B.J., Weng Y.M. (2011). Quality preservation of commercial fish balls with antimicrobial zein coatings. J. Food Qual..

[106] Alkan D., Aydemir L.Y., Arcan I., Yavuzdurmaz H., Atabay H.I., Ceylan C., Yemenicioglu A. (2011). Development of flexible antimicrobial packaging materials against campylobacter jejuni by incorporation of gallic acid into zein-based films. J. Agric. Food. Chem..

[107] Kashiri M., Cerisuelo J.P., Domínguez I., López-Carballo G., Hernández-Muñoz P., Gavara R. (2016). Novel antimicrobial zein film for controlled release of lauroyl arginate (lae). Food Hydrocoll..

[108] Vahedikia N., Garavand F., Tajeddin B., Cacciotti I., Jafari S.M., Omidi T., Zahedi Z. (2019). Biodegradable zein film composites reinforced with chitosan nanoparticles and cinnamon essential oil: Physical, mechanical, structural and antimicrobial attributes. Colloids Surf. B Biointerfaces.

[109] Giteru S.G., Oey I., Ali M.A., Johnson S.K., Fang Z. (2017). Effect of kafirin-based films incorporating citral and quercetin on storage of fresh chicken fillets. Food Control.

[110] Jia F., Wang J.J., Huang Y., Zhao J., Hou Y., Hu S. (2021). Development and characterization of gliadin-based bioplastic films enforced by cinnamaldehyde. J. Cereal Sci..

[111] Balaguer M.P., Lopez-Carballo G., Catala R., Gavara R., Hernandez-Munoz P. (2013). Antifungal properties of gliadin films incorporating cinnamaldehyde and application in active food packaging of bread and cheese spread foodstuffs. Int. J. Food Microbiol..

[112] Bahrami Z., Pedram Nia A., Saeidi-Asl M., Armin M., Heydari-Majd M. (2022). Evaluation of antimicrobial properties of gliadin nanofibers containing zataria multiflora boiss essential oil and its effect on shelf-life extension of smoked salmon fish fillet. Res. Innov. Food Sci. Technol..

